# Emergent Risk Group-4 (RG-4) Filoviruses: A paradox in progress

**DOI:** 10.6026/97320630019829

**Published:** 2023-08-31

**Authors:** John T Sinnott, Kami Kim, Charurut Somboonwit, Conor Cosnett, David Segal, Paul Shapshak

**Affiliations:** Division of Infectious Diseases and International Health, Department of Internal Medicine, Morsani College of Medicine, Tampa, Florida 33606. USA; Wolfram Research Inc., Champaigne, Illinois 61820 USA; College of Health Sciences and Public Policy, Walden University, Minneapolis, Minnesota 55401 USA

**Keywords:** Filo*viridae*, Ebola, Sudan, Zaire, Bundibugyo, Tai forest (formerly Côte d'Ivoire), Reston, Bombali, Marburg, Ravn, Cueva, Thamno, and Stria viruses, World Health Organization (WHO) Risk Group 4 (RG-4) virus pathogens, Biosafety Laboratory (BSL)-4, emergent virus, global warming, ecology, vector, reservoir, humans, monkeys, bats, rodents, sexual risk, health-care setting, virulence, paradox, biodefense, Wolfram Mathematica, ChatGPT, NIH, NIAID, CDC

## Abstract

Filoviruses, categorized as World Health Organization (WHO) Risk Group 4 (RG-4) pathogens, represent significant global health risks due to their extraordinary virulence. The Filoviridae family encompasses Ebola strains such as Sudan, Zaire, Bundibugyo, Tai Forest (formerly known as Ivory Coast), Reston, and Bombali, in addition to the closely related Marburg and Ravn virus strains. Filoviruses originated from a common ancestor about 10,000 years ago and displayed remarkable consistency in genetic heterogeneity until the 20th century. However, they overcame a genetic bottleneck by mid-century. Paradoxically, this resulted in the emergence of boosted virulent strains from the 1970's onward. Filovirus research is included in the NIAID Biodefense Program and utilizes the highest level specialized protective laboratories, Biosafety Laboratory (BSL)-4. The spread of Filoviruses as well as other RG-4 pathogens within Africa poses a significant health threat increasingly both in Africa and out of Africa.

## Background:

Viruses classified under the World Health Organization's (WHO) Risk Group 4 (RG-4) pose a substantial threat to global health, as their rate of spread is currently on the rise. The increased spread is attributable to human-influenced social, economic, and environmental factors. Within this risk group, one notable family is the Filoviridae, which encompasses various strains of the Ebola virus, namely Sudan, Zaire, Bundibugyo, Tai Forest (previously referred to as Cote d'Ivoire), Reston, and Bombali, as well as the closely related Marburg and Ravn virus strains. The Ebola virus is notorious for igniting aggressive epidemics, with prominent acute hemorrhagic illness and also a post-Ebola syndrome. Case fatality rates (CFR) associated with Ebola infections is strikingly high, varying between 25-90%, underscoring the urgent need for comprehensive measures to curb its spread [1, 2,3,4]. The complexities of risks and reservoirs for Filoviruses are under investigation. Bats and humans are virus reservoirs. Infection risks include exposure to bats. Human sexual transmission is also observed and confirmed up to 500 days post-infection. The Ebola virus genome undergoes significant sequence variation. Thus there are restrictions in the use of highly specific quantitative real-time polymerase-chain-reaction (qRT-PCR) assays that are effective for their detection and quantification. A minimum of two separate genome target sequences are used to increase qRT-PCR reliability and minimize false negatives. Additionally, sequence variation places limitations on vaccine development. Similarly, sequence variation must be addressed specifically to produce highly effective and specific vaccines. [4, 5, 6, 7, 8] Of the Ebola virus strains mentioned, Reston and Bombali are not as yet known to cause disease in humans. In 1989, Reston Ebola was isolated from monkeys in Reston, Virginia (USA), which had been imported from the Philippines. This virus caused outbreaks in non-human primates in Pennsylvania and Texas (USA) and in Sienna (Italy). Although investigators became infected with Reston Ebola, they did not become ill. In addition, this virus was subsequently isolated from sick pigs in the Philippines in 2008, where animal caretakers became seropositive but also did not become ill. Epidemiologically, these strains were traced to the Philippines via infected animal commerce. More recently in 2018, the Bombali Ebola virus was isolated from bats in Sierra Leone. [5]

## Ebola Ecology in Africa:

Ebola Virus Disease (EVD), first identified in 1976 in the Democratic Republic of the Congo (formerly Zaire), has since seen sporadic outbreaks throughout West and Central Africa, notably in countries such as Gabon, DRC, Sudan, Cote d'Ivoire, and Uganda. To predict and mitigate the truly existential threat posed by Ebola and its cognate Marburg virus infections in Central Africa, Ecologic Niche Modeling (ENM) was employed. ENM is a technique modeling insightful biogeographical as well as ecological predictions. This work incorporated data from 19 peer-reviewed studies spanning eight countries, examining variables encompassing natural reservoirs of Filoviruses. The spectrum of reservoirs includes humans, non-human primates such as gorillas, chimpanzees, monkeys, bats, rodents, and arthropods. Unanticipated, some plant virus species form part of this diverse list (cf. Figure 2.) [9]

**Figure 1:**
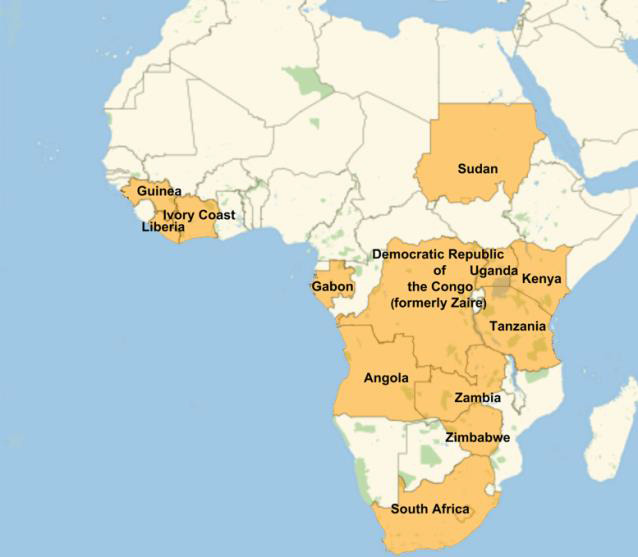
Map of Africa with Filovirus infected countries labeled [4, 5, 11].

Risk factors contributing to the spread of these agents were also considered. These include occupational hazards such as mining, common practices like travel, attending social gatherings, personal contacts, and rarely sexual exposure. Moreover, certain cultural practices can facilitate virus transmission to humans from other species. This cross-species transmission, also known as a 'spillover' or 'jump', could arise from activities including keeping pet primates and monkeys, hunting and consuming bushmeat (and carrion), attending social events, residing in communal dwellings, and camping. Finally, studies also examined additional ecological and socioeconomic parameters. These included contrasting ecological environments such as forests versus savannahs, urban versus rural settings, and modes of transportation - local residential versus highway travel. Factors such as these could significantly influence the transmission dynamics and geographical spread of the Ebola and Marburg viruses [5,9, 10]. Figure 1 shows countries within which Filoviruses have been detected. (Note that not all the indicated countries are contiguous.

Marburg and Ravn viruses are related to Ebola and also are spreading. During 2023, two outbreaks occurred in Equatorial Guinea and Tanzania due to Marburg virus. It's reservoir is the Egyptian fruit bat. Work is being done to ascertain if the two outbreaks were separate virus jumps or epidemiologically linked. [12,^13^] Genome sequence clock evolutionary studies demonstrated that Ebola and Marburg viruses diverged a few thousand years ago. Since then, both viruses exhibited relatively stable heterogeneity. Sequence bottlenecks occurred next, and fewer strains were extant in the 1900's. However, paradoxically, increased pathogenicity as well as strain diversification occurred in animals and humans, prior to the outbreaks of the 1970s. [13,14]

## Phylogeny of Related Virus Families:

**Figure 2:**
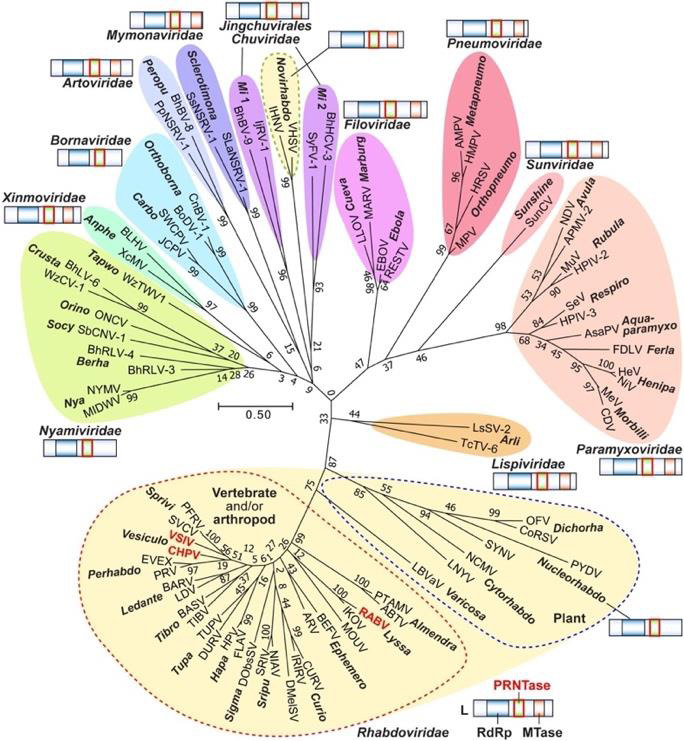
Phylogenetic relationships within the order, Mononegavirales. The phylogenetic relation of Filoviridae, with other virus families includes Rhabdoviridae, Paramyxoviridae, Lispiviridae, and Bornaviridae Nyamiviridae, Sunviridae, Xinmoviridae, Artoviridae, Chuviridae, Pneumoviridae, and several members of a Plant virus group. This image is reproduced with permission from elsewhere [17].

As mentioned, Ebolavirus and Marburgvirus genera are members of the Filoviridae family. Carrol et al performed Bayesian coalescent phylogenetic analysis of 97 complete virus genomes. Virus molecular evolution rates (nucleotide substitutions/site/year) vary from 0.46 x 10^-4^ to 8.21 x 10^-4^ for Sudan and Reston ebolaviruses, respectively. In greater detail, about 10,000 years ago, the Filoviridae family had a common ancestor. More recently, the Marburg virus group shared a common ancestor about 700 years ago and the Ebola virus group shared a common ancestor and the Ebola virus groups shared common ancestors 850 years ago. [15] The Marburg and Ebola virus genera are members of the Filoviridae family of Filoviruses, which along with ten other families of viruses, make up the Mononegavirales order (Figure 2). There is evidence suggesting that Mononegaviruses may have divergences which date back tens of thousands to millions of years based on the existence of viral gene fragments detected in mammalian genomes. [15,16] Figure 2 shows the relation of Filovirus family, Filoviridae, with cognate family viruses within the order, Mononegavirales. [17]

## Ebola out of Africa:

In addition to Filovirus infections expansion in Africa, including Angola, South Africa, Cote d'Ivoire, Guinea, Sierra Leone, the Democratic Republic of the Congo (formerly Zaïre), Uganda, Kenya, and Sudan, Filovirus infections spread out of Africa. [18] The widening expansion of Ebola in Africa is in great part associated with increased risks, including changes in factors such as weather, climate, ecology, economy, and socio-demography. Further research indicates that these changes have also reached China and other countries. [19] For example, in Thailand, there is an increased risk for Ebola virus infection involving ecological locales and regions. From 2011 to 2014, studies were done in five provinces in Thailand. Direct analysis of saliva, serum, and urine were analyzed for Ebola virus by PCR and IgG ELISA. More than 1,300 specimens were analyzed from 26 bat species and one Macaque species. Although no positives were detected, continued testing was recommended because of the ecological risk factors mentioned. Disease surges are anticipated and continued testing is advised. [20] Large Marburg virus disease outbreaks occurred in 1967 in Marburg and Frankfurt (Germany) and in Belgrade (Serbia), which led to disease recognition. The infection source was African Green monkeys that were imported from Uganda. [15, 20, 21] Captive macaques were fatally infected with Ebola in the Philippines in 1992. Ebola virus RNA was detected in bats in Spain, 2004, in bats in China, 2015. Possibly PCR-negative bats can be infectious. The literature supports the need for continued Ebola virus and disease surveillance in animals and humans, within and outside Africa. Additional geographical areas that require such studies of the emergent Ebola and Marburg viruses as soon as possible, with the emergency, include the Americas, Europe, Asia, and Australia. [20, 22,23,24,25, 26,27,28,29,30, 31] Briefly, several countries that experienced Filovirus infections include: Spain in 2011, Hungary in 2016, Germany in 1967, Yugoslavia in 1967, China in 2018 and 2019, Philippines in 1989, 1990, 2008, and 2015, and the USA in 1990. [18]

## Pathogenicity Paradox:

Standard virus virulence theory states that virulent viruses become less virulent over time. However, the history to date of EVD does not conform to that theory. This is a challenging current central question that is being addressed. [32, 33]

## Conclusions:

Filoviruses are among the planet's most virulent viruses and are spreading. In actuality, five genera are distinguished among the family Filoviridae: Ebola virus, Marburg virus, Cuevavirus, Thamnovirus, and Striavirus. Continued research and clinical studies are needed. However, major restrictive bottlenecks to accomplish these goals include the extreme dangers posed by the Filoviruses themselves and the social conflicts, terrorism, wars, suspicions, and violence to strangers on the part of some risk/susceptible populations. Clinical diagnostic methods, research, and vaccine development require advances in the pharmaceutical pipeline as well. [2, 3, 7, 9, 10, 34, 35]
